# Effects of artificial milk on growth performance and intestinal barrier function in suckling piglets: a review

**DOI:** 10.3389/fvets.2026.1967552

**Published:** 2026-10-01

**Authors:** Guangyao Song, Jingrong Feng, Han Yuan, Huijin Jia, Wanghong Zhang, Sumei Zhao, Junhong Zhu

**Affiliations:** 1 Yunnan Provincial Key Laboratory of Animal Nutrition and Feed, Faculty of Animal Science and Technology, Yunnan Agricultural University, Kunming, China; 2 College of Animal Husbandry and Veterinary Medicine, Yunnan Vocational College of Agriculture, Kunming, China

**Keywords:** artificial milk, growth performance, intestinal barrier function, intestinal health, suckling piglet

## Abstract

With the continuous improvement of genetic selection in modern swine production, litter size has steadily increased. However, due to the limited capacity of sows to enhance milk production, some piglets experience insufficient milk intake and restricted growth during the lactation period. As an important supplement to sow milk, artificial milk has been widely used in both production practice and scientific research. This review summarizes the effects of artificial milk on the growth performance and intestinal health of suckling piglets. It analyzes how insufficient milk yield and the nutritional composition of sow milk affect piglet growth, and then discusses the positive roles of artificial milk in alleviating the lactational burden on sows, mitigating environmental stress, and improving weaning weight and survival rate of piglets. Furthermore, the potential mechanisms by which artificial milk promotes intestinal development and enhances growth performance are examined from the perspectives of intestinal morphology, digestive function, and the mechanical, mucus, microbial, and immune barriers of the gut. This review aims to provide a theoretical basis for the management of piglets born to sows with extremely large litters.

## 1 Introduction

Through advances in genetic selection and management practices, modern swine production has successfully bred sows with shorter weaning-to-estrus intervals and larger litter sizes (1). Although milk yield increases with litter weight and reaches its maximum at a litter size of 14 piglets (2), piglets that fail to consume sufficient milk face a higher risk of mortality due to inadequate energy reserves, poor thermoregulatory capacity, and insufficient colostrum intake (3). Because piglet growth and development rely heavily on sow milk (4), insufficient milk supply or intensified teat competition—particularly in larger litters and hyperprolific sows—severely restricts piglet growth (5). To address this issue, artificial milk has attracted considerable attention in both research and practice as a nutritional strategy to supplement or replace sow milk. Typically reconstituted with water, artificial milk is used to partially or fully replace sow milk as a nutritional source for feeding piglets, especially in cases of insufficient milk production or large litters. It is designed to provide energy, protein, fat, vitamins, and minerals comparable to those in sow milk to support piglet growth and development (6). The application of artificial milk can be broadly categorized into two scenarios. In the first, piglets remain with the sow and have ad libitum access to both sow milk and artificial milk, which helps mitigate within-litter competition, improve nutrient intake of disadvantaged piglets, enhance litter uniformity, and compensate for the dilution of milk distribution (3, 7–9). In the second scenario, piglets are separated from the sow, and artificial milk serves as their primary nutritional source, making it easier to observe systematic differences in growth, intestinal development, microbiota, and metabolism between artificial milk and sow milk (6, 10, 11). Feeding piglets with artificial milk has become a common practice in the swine industry, and its effects on growth performance and intestinal health have drawn increasing research attention. This review aims to elucidate the impact of artificial milk on piglet growth and intestinal barrier function.

## 2 The effects of sow milk on the growth and development of piglets

Milk, secreted by the mammary gland, serves as the ideal food for newborn mammals, contributing to immune protection and promoting organ development in neonates (12). In addition to water, sow milk is primarily composed of three major components: protein, lactose, and fat (13). During lactation, sow milk can be divided into three stages—colostrum, transitional milk, and mature milk—each exhibiting significant compositional differences (14). As shown in Table 1, although the levels of crude protein (CP), ether extract (EE), and lactose vary among different breeds of sows, within the same breed, the crude protein content in milk gradually decreases from colostrum to transitional milk and then to mature milk. Milk fat is relatively low in colostrum, increases during the transitional phase, and then slightly declines before stabilizing in mature milk. In contrast, lactose content progressively increases from colostrum through transitional milk to mature milk. If colostrum is regarded as a composite product of immunoglobulins and milk, the gradual transition from colostrum to mature milk can be well explained: before parturition, the mammary gland begins to uptake immunoglobulins from the blood and secrete them into colostrum; after parturition, the closure of tight junctions between mammary epithelial cells halts the transport of immunoglobulins into milk, thereby terminating colostrum synthesis. At this point, the total protein content in milk decreases significantly due to the rapid decline in immunoglobulin concentration. The synthesis of fat and lactose is primarily initiated after parturition and gradually intensifies. As lactation proceeds, colostrum is continuously expelled while milk is persistently synthesized, leading to the progressive dilution of the original colostrum components. Consequently, the protein level in milk gradually declines, whereas the proportions of fat and lactose increase (15–18). Sow milk plays a crucial role in improving piglet performance (19) and stimulating protein synthesis in the visceral organs and skeletal muscles of neonatal piglets (20). However, when milk yield (4) and quality (19) decline, both milk and the sow may negatively affect the growth and development of piglets.

**Table 1:** The composition of crude protein, crude fat, and lactose in the milk of sows from different breeds.

Type of milk	Sow breeds	CP %	EE%	Lactose %	References
Colostrum	Landrace × Large White	14.56	5.98	3.19	(131–134)
Colostrum	Large White × Landrace	15.44	4.90	3.60	(135, 136)
Colostrum	Large White	12.55	4.26	4.09	(137)
Colostrum	Landrace	8.93	7.98	2.51	(138)
Colostrum	Ningxiang Pig	18.17	4.59	3.73	(139)
Colostrum	Iberian pig	15.8	3.76	2.34	(30)
Colostrum	PIC	15.02	5.12	2.34	(140)
Transitional milk	Landrace × Large White	7.20	7.10	4.30	(132)
Transitional milk	Landrace	6.57	9.23	3.97	(138)
Mature milk	Landrace × Large White	5.42	6.51	5.18	(131–134)
Mature milk	Large White × Landrace	4.57	6.79	5.47	(135, 136)
Mature milk	Large White	5.1	6.06	5.60	(137)
Mature milk	Landrace	5.96	7.81	5.44	(138)
Mature milk	Iberian pig	5.31	5.91	5.71	(30)
Mature milk	Camborough	4.83	6.73	5.53	(141)

### 2.1 The impact of insufficient milk production on the growth and development of piglets

The growth and development of suckling piglets are heavily dependent on sow milk. Using deuterium dilution techniques to measure actual milk intake, it has been estimated that approximately 4 grams of milk are required for each gram of piglet body weight gain (21, 22). However, the milk production of sows is often insufficient to support the rapid growth of their piglets (4). Studies have shown that piglets achieve the highest daily weight gain during the second week of lactation (23), while the lightest and heaviest piglets exhibit a decline in growth rate during the third and fourth weeks (24). This phenomenon is closely related to the lactation performance of the sow. The average peak milk yield of sows is 9.23 kg, occurring at approximately 18.7 days postpartum, after which milk production begins to decline (25). Sows are unable to produce enough milk to sustain the optimal growth of their entire litter, and this insufficiency becomes apparent as early as 8 to 10 days of age (24). As litter size increases, the sow’s inability to consume sufficient nutrients leads to a linear decrease in the growth rate of individual piglets. This effect is particularly pronounced during the later stages of lactation: when litter size increases from 6 to 14 piglets, the average growth rate drops from 309 g/day to 199 g/day (2). Moreover, an increase in litter size is associated with a lower average birth weight, greater within-litter birth weight variability, and a higher proportion of low-birth-weight piglets (26). It has been reported that each additional piglet in a litter reduces the average birth weight by 40 g, increases the coefficient of variation in birth weight within the litter by 0.76%, and raises the proportion of piglets with a birth weight below 800 g by 1.48% (27). Some piglets, due to insufficient vitality or an inability to compete effectively with larger littermates for access to teats, fail to consume adequate amounts of milk (19).

In summary, in modern high-yield systems, the mismatch between litter nutritional requirements and milk production is the main constraint limiting the growth of suckling piglets. Milk deficiency becomes apparent in the second week of lactation and is exacerbated by competition within the litter, with the greatest cost for piglets with low birth weight. Increasing the actual milk intake per piglet may be the most direct way to protect early growth.

### 2.2 Effects of sow milk nutrient composition on the growth and development of piglets

Piglet growth is largely dependent on milk intake and milk protein concentration, with higher milk protein levels and greater milk consumption leading to increased weight gain during the second and third weeks of lactation (22, 28). This is likely because each gram of body protein binds approximately 4.2 grams of water, meaning that weight gain in piglets primarily reflects the retention of protein and water rather than ash or fat (29). Studies have shown that the growth rate of suckling piglets may not be limited by insufficient nutrient supply from milk, but rather by the extremely low efficiency of utilizing milk protein and energy (30). When milk protein content reaches 4.9%, piglets exhibit greater weight gain and lower body fat levels (22, 31), indirectly suggesting that insufficient milk protein may impair piglet growth (32). Lactose, as a carbohydrate in milk, serves as an energy source, participates in thermogenesis, and provides energy for protein retention, thereby positively influencing piglet growth rate (33). Milk fat is primarily composed of triglyceride fatty acids, which already exert beneficial effects during the fetal period and later serve as a major energy source for piglets through milk, influencing the development and function of various organs (34, 35). Research has found that differences in fat and fatty acid levels between sow colostrum and mature milk affect piglet rearing outcomes: when milk fat and unsaturated fatty acid content are elevated, piglets achieve optimal body weight, growth rate, and lower mortality (36). However, other studies indicate that the rate of nutrient deposition is highly correlated with total milk volume and nutrient intake, rather than with the percentage composition of milk constituents per se, and that increasing total consumable milk volume often explains growth improvements more effectively than fine-tuning component percentages (22).

Sow diet also influences milk production and consequently affects the growth and development of piglets. Dietary protein levels impact milk quality, as up to 70% of dietary protein is utilized by lactating sows for milk protein synthesis (37). Adequate protein intake can improve piglet growth rates and reduce body weight loss (38). However, under low dietary protein levels, the effects of reduced protein content on total solids, fat, and protein in milk become more pronounced. Lowering dietary protein concentration not only decreases milk yield but also reduces the protein and fat content in milk, thereby lowering total solid content (39). Furthermore, dietary supplementation with additives can improve sow milk composition. For instance, arginine increases milk fat content (40); laminarin improves the fatty acid profile of sow milk (41) nucleotides elevate nucleotide concentrations in milk (42); and yeast culture enhances lactose content in colostrum and crude protein content in mature milk (43). These additives effectively improve piglet growth performance by enhancing milk composition. Additionally, supplementing lactation diets with high-energy ingredients such as fats or oils can increase energy intake and energy output in milk, thereby reducing sow body weight loss and improving piglet weight gain (44). However, some studies indicate that fat supplementation has no significant effect on birth litter weight (*p* = 0.40) or weaning litter weight (*p* = 0.46), although it tends to increase total litter size (*p* = 0.07), with no significant impact on the number of live-born piglets (*p* = 0.90) or piglet weaning survival rate (*p* = 0.48) (45). These findings highlight the complexity of the effects of sow diet on suckling piglets.

In summary, the growth of suckling piglets is jointly determined by the sow’s milk yield and the absolute supply of protein, lactose, and fat in the milk. Milk protein can contribute to weight gain by increasing protein deposition and binding with water; lactose provides energy for protein deposition and heat production; and unsaturated fatty acids in milk fat are beneficial for improving piglet survival rates. Although dietary intervention in sows can alter milk composition, some experiments have shown that total edible milk volume is a better explanation for growth improvement than fine-tuning the composition ratio. Therefore, increasing the total intake of milk and optimizing milk quality are beneficial for improving piglet growth performance.

## 3 The substitution effect of artificial milk for sow milk

The weaning weight of piglets is closely related to the amount of milk they consume during lactation, as milk serves as the primary source of nutrition during the first three weeks of life (46). However, as sow productivity improves and litter size continues to increase, milk production often fails to keep pace, resulting in a reduced volume of milk available per piglet (4). This reduction in milk intake negatively affects piglets, leading to higher pre-weaning mortality (47), and lower average daily gain before weaning (2). In particular, under conditions of hyperprolific sows or large litters, the sow’s milk yield may be insufficient to meet the needs of the entire litter, often resulting in underdeveloped piglets, intensified within-litter competition, and even piglet mortality (5). Artificial milk, available in powder or liquid form, is typically composed of dairy products such as skim milk powder, whey powder, and whey protein concentrate, along with corn, soybean meal, barley, soybean oil, amino acids, vitamins, and mineral elements. Some products also contain probiotics (Table 2). It serves as an effective alternative or supplement in situations where sows have insufficient milk production or limited teat availability, thereby improving weaning performance and reducing within-litter weight variation, while also alleviating the high lactation burden on sows. Supported by extensive experimental studies and commercial data, artificial milk has been widely adopted in practice (24, 48).

**Table 2:** Ingredient composition and nutrient levels of the artificial milk formula.

Chemical composition ingredient composition	Formula A (81)	Formula B (76)	Formula C (142)	Formula D (143)
Barley (%)	5			
Maize (%)	23.1			
Soybean meal (%)	14.342			
Soya full fat (%)	13.081			
Skim milk powder (%)	12.5	17.61	63	
Coco fat filled whey (%)		42		
Milk replacer (%)				37.50
Whey permeate (%)	20	8.29		
Soy protein concentrate (%)		10		
Isolated soy protein (%)				9.20
Soya oil (%)	8.5		19.9	
Whey protein concentrate (%)		7		
Whey permeate (%)		8.29	15	39.59
Lactose (%)				6.40
Spray dried blood plasma (%)		4		
Vitamin and mineral premix (%)	0.3	1.8	1	0.2
Limestone flour (%)	0.7		1	
Dicalciumphosphate (%)		0.32		
Lysine HCl (%)	0.622			
Lysine 78% (%)				0.57
DL-Methionine (%)	0.362	0.31	0.1	
Methionine 99%				0.36
L-Threonine (%)	0.364			
Threonine 98% (%)				0.26
L-Valine (%)	0.129			
Citric acid (%)		0.3		
L-Tryptophan L- (%)	0.14	0.08		
Tryptophan 10% (%)				0.90
Ronozyme HiPhos GT (%)	0.01			
Salt (%)	0.3			
Sugar (%)				3.50
Glucose (%)				1.00
Sweetener (%)				0.02
Mono di-calcium phosphate (%)	0.55			
Organic acid (%)				0.50
probiotics (CFU/g)				1 × 10^9^
Chemical composition	Formula A (81)	Formula B (76)	Formula C (142)	Formula D (143)
CP (%)	19.13	24.9	22.6	20.56
EE (%)	12.915	20.5	20	6.03
Lactose (%)			46	50.67
Ash (%)	5.55		7.1	6.46
Ca (%)	0.819		1.4	0.69
Phosphorus (%)			0.7	0.59
Digestible P (%)	0.462			
Net energy (MJ/kg)	12.06	15.48		
Digestible energy (kcal/kg)				3,948
SID lysine (%)	1.62			
Lysine (%)			1.7	1.88
Digestible lysine (%)		1.82		

### 3.1 Reducing the reproductive burden on sows

The nutritional requirements of sows increase substantially with litter size, and in theory, nutrient supply should exceed the average by a certain margin. However, this demand is often difficult to meet, exacerbating supply bottlenecks during mid-to-late lactation for sows with higher nutritional needs and leading to the mobilization of body fat and protein reserves (44, 49, 50). Although sows can tolerate a loss of up to 9 to 12% of pre-existing body protein mass during lactation without adversely affecting piglet growth or ovarian function, a loss exceeding 16% may impair subsequent reproductive performance (51, 52). Backfat loss resulting from body fat mobilization is also negatively correlated with reproductive performance; sows that experience greater backfat loss during lactation tend to have lower pregnancy rates and require a longer interval to return to estrus (53). In contrast, body protein loss impairs piglet growth performance. From day 20 of lactation to weaning, the growth rates of piglets from sows in low, moderate, and high protein loss groups changed by +13%, +7%, and −11%, respectively, relative to the average growth rate during the first 20 days of lactation, with significant differences among treatment groups (*p* < 0.02). Sows with the greatest protein loss exhibited the largest reduction in piglet growth rate (*p* < 0.02) (52).

Providing artificial milk to suckling piglets can reduce their dependence on sow milk and alleviate the sow’s lactation burden (48). In a commercial pig farm case study, sows with oversized litters were provided with artificial milk from day 2 after birth until weaning. The results showed that sows in the artificial milk supplementation group successfully reared a higher number of piglets compared to the control group (13.5 vs. 12.4), and the total weaning weight per litter was also significantly greater (104.9 kg vs. 96.7 kg), while no significant differences were observed in sow body weight, backfat thickness, or body condition score (54). Similarly, a study on hyperprolific sows (litter sizes of 14 vs. 17 at birth) with or without artificial milk provision found that the supplemented group had significantly higher piglet weaning survival rates and litter weights, whereas changes in sow body weight loss and backfat thickness did not differ significantly between the two groups (3). These findings indicate that when sows have insufficient milk production or are under excessive lactation demands, artificial milk can effectively substitute part of the functions of sow milk, supporting piglet growth without increasing the physiological burden on the sow.

Therefore, when the litter size exceeds the lactation capacity of sows, artificial milk can replace a portion of breast milk intake without consuming additional body protein or backfat of sows. This indicates that artificial milk is not only a measure to improve the nutrition of piglets, but also beneficial to protect the subsequent reproductive performance of sows.

### 3.2 Counteracting stress

Nutritional intervention can improve sow body condition and increase piglet birth weight to some extent (55). However, maternal stress may negatively affect piglet growth performance (56). Quantile regression analysis revealed that high temperatures exert a stronger influence on sows with lower feed intake during mid-to-late lactation, making them more susceptible to insufficient nutrient supply (57). Under heat stress, sows reduce feed intake by approximately 38%, experience greater body weight loss, and exhibit decreased litter survival rate, litter weight, and litter size. Relying solely on sow milk production may compromise reproductive efficiency, whereas the use of artificial milk mitigates these adverse effects (58). Milk synthesis during lactation imposes a substantial metabolic burden on sows, and oxidative stress during this period further reduces milk yield, which in turn impairs piglet growth (56).

Supplementing piglets with artificial milk has also been shown to be beneficial in mitigating stress. In a study spanning 171 litters over 3.5 years, the provision of artificial milk significantly increased weaning weight (6.4 kg vs. 5.6 kg; *p* < 0.001) and total litter weight (60.9 kg vs. 52.2 kg; *p* < 0.001) (59). The study further indicated that seasonal and environmental factors influence the effective intake of artificial milk, with piglets consuming significantly less during cold seasons compared to warm seasons. Similarly, under heat stress conditions, the growth-promoting effect of artificial milk during the lactation period becomes more evident. In trials conducted under high-temperature or summer conditions, supplementation with artificial milk led to higher weaning weights (5.37 kg vs. 4.82 kg; *p* < 0.01), improved average daily gain during lactation, and reduced piglet mortality (60). Moreover, feeding artificial milk may help piglets achieve optimal weaning weights (61).

In summary, stress and seasonal low temperatures can all reduce milk production or decrease the willingness of piglets to consume supplemental milk. Sows are most vulnerable to impaired feeding and lactation under high temperature conditions. Piglets in the artificial milk group have greater weight gain and lower mortality rates, indicating that artificial milk can mitigate the adverse effects of the environment and enhance piglet production efficiency.

### 3.3 Promotion of growth and development in piglets

Providing artificial milk to suckling piglets can also improve their weaning weight, litter weaning weight, and weaning number to varying degrees, reduce piglet mortality, and may have a positive impact on the post-weaning weight of piglets. An analysis involving 150 litters and 1,709 piglets revealed that liquid milk supplementation reduced the coefficient of variation in weaning weight from 25.3% to approximately 20.3% (24). When evaluating the impact of piglet birth weight and liquid milk substitutes on growth performance during lactation, it was found that for low birth weight (LBW) piglets, feeding supplemental milk powder during lactation resulted in heavier piglet weights at weaning (6.6 vs. 5.7 kg; *p* < 0.001), and tended to increase the number of weaned piglets (11.4 vs. 10.9 per litter; *p* = 0.10) (62). For super-high-yield sows standardized to deliver 14 and 17 live births per litter, artificial milk increased the litter weight before weaning, indicating that when the number of piglets exceeds the number of functional teats, artificial milk can reduce the impact of decreased growth performance caused by insufficient access to breast milk (3). Additionally, by adding odd-chain fatty acids to artificial milk, piglet growth can also be improved. Compared to feeding breast milk, artificial milk supplemented with odd-chain fatty acids significantly increased daily weight gain of piglets (63). Supplementation with artificial milk also had beneficial effects on the number of weaned piglets and mortality rates. One study reported that the number of weaned piglets in the artificial milk group was 13.5, significantly higher than the 12.4 observed in the control group (*p* < 0.05). Although average weaning weight and average daily gain did not differ between groups, total litter weight at weaning was significantly greater in the artificial milk group (104.9 kg vs. 96.7 kg; *p* < 0.001) (54). Increased body weight enables earlier weaning, and the sow feed saved as a result of earlier weaning is estimated to range between 15 and 25 kg (59).

The effect of artificial milks on post-weaning growth in piglets is a key focus of research. Supplementing with artificial milk during the five days prior to weaning significantly increased average daily gain from birth to weaning and resulted in significantly higher weaning weights. This advantage persisted into the post-weaning period: from week one to week four after weaning, the pre-weaning artificial milk supplementation increased both average daily feed intake and average daily gain, and improved feed conversion efficiency (64). In one study evaluating the effects of birth weight and liquid artificial milk provision during lactation on growth performance up to slaughter weight, it was found that although artificial milk had no significant impact on growth performance from weaning to slaughter, piglets fed artificial milk reached a body weight of 110 kg three days earlier than those that were not (62). Other research has shown that providing artificial milk from early lactation may effectively increase weight gain in low-birth-weight piglets. Although the growth of piglets during the nursery and growing phases was not affected by the feeding regimen, body weights at day 55 and day 135 were highly correlated with weaning weight (65). Previous experiments have also found that weaning weight significantly influences subsequent growth performance, with heavier piglets at weaning exhibiting greater weight gain during both the nursery and finishing periods (66). Furthermore, well-fed gilts experienced higher growth rates and reached sexual maturity slightly earlier (67). These findings suggest that increasing weaning weight through the use of artificial milk can enhance post-weaning growth rates, thereby improving overall production efficiency.

In summary, artificial milk can enhance daily weight gain, weaning weight, and uniformity in promoting the growth and development of piglets. In large litters, it can also increase the number of weaned piglets and potentially improve their subsequent growth performance. Additionally, it can reduce the lactation burden on sows, decrease piglets’ dependence on breast milk, mitigate the impact of stress on piglets, and improve their growth performance.

## 4 Artificial milk enhances growth performance in piglets by improving intestinal health

The intestine of neonatal piglets undergoes a critical window of rapid development and functional maturation, characterized by high rates of epithelial turnover, the establishment of the mucosal barrier and mucosal immune tolerance, and the concurrent colonization of gut microbiota whose metabolites—such as short-chain fatty acids (SCFAs) and bile acid derivatives—collectively shape intestinal homeostasis (68, 69). Within the first 6 h after birth, total intestinal weight, mucosal weight, and protein content increase by 58%, 80%, and 126%, respectively (70). Villus height and width, crypt depth, and mucosal thickness in the small intestine increase from day 0 to day 3, but decrease from day 3 to day 14 (71). Nutritional status has been shown to significantly influence intestinal mucosal growth and function. Porcine colostrum contains a trypsin-labile factor that enhances intestinal alkaline phosphatase activity in neonates (72), and intestinal alkaline phosphatase, expressed in villus-associated enterocytes, is essential for maintaining gut homeostasis (73). Intestinal health is closely associated with growth performance. Compared with normal birth weight piglets, low birth weight piglets exhibit poorer growth performance (a 29.9% reduction), lower mRNA levels of claudin-1 (a 63.6% reduction), and higher jejunal concentrations of interleukin-1 (IL-1) (an 18.8% increase) (74). As a supplement or substitute for sow milk, artificial milk not only alters the form of nutrient supply but also reshapes the intestinal environment, thereby improving growth performance in piglets (75). Its effects on intestinal health are primarily reflected in aspects such as intestinal morphology, digestive function, and intestinal barrier integrity (69, 76).

### 4.1 Effects on intestinal morphology and digestive function in piglets

Feeding artificial milk may stimulate the maturation of the digestive system in piglets (77). Intestinal morphology is one of the primary indicators of gut health in swine (78). Compared with the control group, piglets supplemented with artificial milk exhibited significantly greater intestinal weight per unit length at 21 days of age, along with increased crypt depth and elevated expression of the cell proliferation marker PCNA. These findings suggest enhanced proliferation of intestinal epithelial cells, increased body weight, improved nutrient absorption capacity, and potentially beneficial effects on the weaning transition (75). In a similar study, the early artificial milk feeding group showed greater jejunal villus height and intestinal weight per unit length than the sow-reared control group, along with improved growth performance, supporting the view that artificial milk promotes intestinal development and enhances growth (79). Compared with suckling piglets, those fed artificial milk exhibited a larger absorptive surface area, higher maltase and sucrase activities, and deeper crypts, all of which contributed to improved nutrient digestion and absorption (80). On day 7 post-weaning, piglets that had received artificial milk supplementation for 11 days showed a 37% increase in jejunal villus height and approximately 180% higher maltase activity compared with the control group, as well as significantly greater average daily gain. These results indicate that milk supplementation promotes intestinal functional maturation, enhances growth performance, and alleviates the negative effects observed during the early post-weaning period (81).

In brief, artificial milk often increases intestinal mass per unit length, villus height, and crypt cell proliferation, and enhances the activities of maltase and sucrase. These morphological and enzymatic changes provide an intestinal explanation for weight gain before and early after weaning.

### 4.2 Effects on intestinal barrier function in piglets

The intestinal barrier comprises the gut microbiota, the overlying mucus layer, the columnar epithelium and extracellular matrix, and the innermost lamina propria, which contains blood and lymphatic vessels, immune cells, and numerous intrinsic and extrinsic nerve endings (82). The use of artificial milk may affect intestinal barrier function at multiple levels (Figure 1).

**Figure 1:**
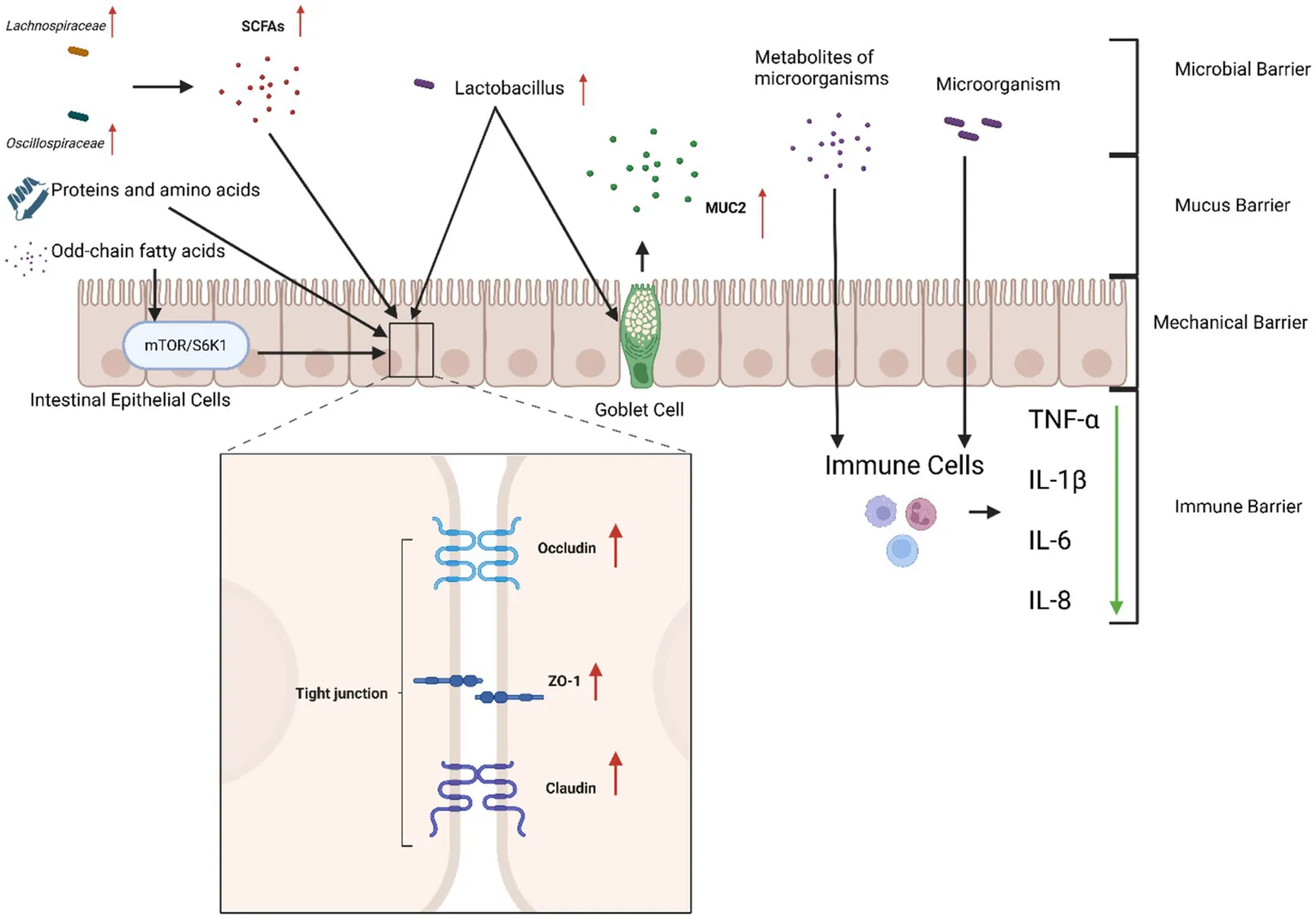
Effect of artificial milk on the intestinal barrier function of piglets. Artificial milk and its nutrients enhance the intestinal barrier function of piglets in multiple ways by improving the gut microbiota structure and metabolites, increasing the expression of tight junction proteins, promoting the secretion of MUC2 by goblet cells, and reducing the expression of pro-inflammatory cytokines in the body.

#### 4.2.1 Mechanical barrier and mucus barrier

Within the intact epithelium, tight junctions serve as critical intercellular connections that support the integrity of the intestinal mechanical barrier (83). Located at the apical region of cell–cell contacts, tight junctions are fundamental structures between epithelial cells. The barrier formed by tight junction proteins effectively prevents the invasion of intestinal bacteria and toxins (84). In contrast, the mucus barrier is primarily composed of a mucus layer secreted by goblet cells, which consists of water, phospholipids, mucins (MUC), secretory immunoglobulin A (IgA), antimicrobial peptides, and various defensive factors (84). This mucus layer plays an essential role in preventing the attachment and invasion of microorganisms and harmful substances, protecting epithelial cells from physical abrasion, and facilitating epithelial cell renewal and differentiation (82, 85).

Dietary interventions can enhance the expression of tight junction protein-encoding genes in the mechanical barrier (86). Artificial milk significantly increases the final body weight and average daily gain in piglets, and improves tight junction protein expression by upregulating the mRNA levels of zonula occludens-1 (ZO-1), occludin, and claudin-1 in the jejunum (87). As one of the proteins in milk, lactoferrin can stimulate the proliferation of crypt cells in newborn piglets. Feeding artificial milk containing bovine lactoferrin during the first 14 days after birth increases the proliferation, depth, and area of the jejunal crypts (88). In addition, there is a synergistic effect among milk proteins. By optimizing the protein composition in the formula (such as lactoferrin, lactalbumin, dietary osteopontin, casein, and its hydrolyzed peptides), the villus height of the duodenum and ileum in piglets can be increased, and the abundance of ZO-1, claudin-1, and occludin can be increased by 1.5 to 3 times, effectively improving the mechanical barrier of the piglet’s intestine (89). The odd-chain fatty acids can also improve the intestinal morphology and function of piglets. The odd-chain fatty acids in artificial milk can increase the ratio of villus height to crypt depth and enhance the protein expression levels of Occludin, Claudin-1, and ZO-1 (63). The mechanism behind this is that after feeding artificial milk containing odd-chain fatty acids, the protein expression levels of Ki67, p-mTOR, and p-S6K1 in the intestine increase, and the mTOR/S6K1 signaling pathway regulates the expression of these tight junction proteins (63). Ki67 is used to evaluate the proliferation of intestinal epithelial cells (90). The activated mTOR/S6K1 signaling pathway can promote the proliferation and migration of intestinal cells (91). Therefore, odd-chain fatty acids can improve intestinal morphology through increased cell proliferation. Functional amino acids are also one of the regulators of the intestinal barrier. Research has shown that arginine is an essential amino acid for young mammals, and a deficiency of arginine may limit the maximum growth of newborn piglets (92, 93). By supplementing arginine in artificial milk, the villus height of the jejunum and ileum of piglets is increased, daily weight gain is enhanced, and the mRNA level of Claudin1 in the jejunum is also increased by 105% (74). Similarly, another study reported that artificial milk reshaped the intestinal microbiota of piglets, significantly increased the mRNA expression of ZO-1, occludin, and claudin-4 in the colon, reduced diarrhea incidence, and improved growth performance (79). Mucin 2 (MUC2), which is produced and secreted by intestinal goblet cells, plays a critical role in protecting the intestinal mucus barrier (94). Artificial milk enhances MUC2 production and secretion by modulating metabolites and the gut microbiota (79), thereby reducing the contact between intestinal epithelial cells and harmful substances and ultimately decreasing the frequency of inflammation (95).

In summary, the nutritional components in artificial milk, such as odd-chain fatty acids and arginine, enhance the intestinal mechanical and mucosal barrier functions of piglets by promoting the proliferation of intestinal epithelial cells, improving intestinal morphology, and upregulating the expression of barrier-related proteins such as ZO-1, Occludin, Claudin, and MUC2.

#### 4.2.2 Microbial barrier

The gut microbiota comprises bacteria, fungi, viruses, archaea, and protozoa, with bacteria accounting for over 70% of this community (96, 97). Most gut bacteria belong to two major phyla: Bacteroidetes and Firmicutes (98). These bacteria enhance host digestive efficiency by breaking down dietary polysaccharides into absorbable compounds (99). They also limit the colonization of pathogenic bacteria in the gut by competing for nutrients (100, 101). Furthermore, the gut microbiota can stimulate host immune responses, thereby preventing pathogen invasion (102).

Dietary changes can alter the gut microbiota composition of suckling piglets (69, 103). Artificial milk can enhance microbial richness by improving the intestinal flora of piglets (104), and through increased microbial fermentation, it inhibits pathogen proliferation and reduces the risk of intestinal inflammation (105). For instance, artificial milk has been shown to improve the gut microbiome composition of piglets, with a significant reduction in the relative abundance of certain bacterial species compared to the control group (87). Another study found that supplementation with artificial milk significantly reduced mortality from postnatal day 5 to day 14 and throughout the pre-weaning period. The fecal microbiota was characterized by an enrichment of short-chain fatty acid (SCFA)-producing genera, such as Lachnospiraceae and Oscillospiraceae, suggesting that artificial milk can also promote the colonization of SCFA-producing bacteria and influence gut microbiota composition (106). Lactic acid bacteria are considered to play a key role in actively protecting the intestinal barrier (107). Artificial milk can increase the abundance of Lactobacillus in the colon, elevate the Firmicutes-to-Bacteroidetes ratio, and thereby improve the gut microbiota composition of piglets (79). Lactic acid bacteria can upregulate the gene expression of tight junction proteins (108) and may enhance mucin production and secretion, thereby strengthening the intestinal barrier and improving gut health (109). However, some studies have reported that the abundance of Enterobacteriaceae in the ileum was higher in the artificial milk group than in the sow milk group, along with lower alpha diversity, suggesting that artificial milk supplementation may delay microbiota maturation (110).

The gut microbiota also contributes to the maintenance of the intestinal barrier through its metabolites. Indigestible carbohydrates can be fermented by bacteria in the distal gut, including the cecum and colon, into short-chain fatty acids (SCFAs) (111), which are essential for preserving intestinal health and alleviating inflammation (112). Studies have shown that piglets fed with artificial milk exhibit elevated concentrations of SCFAs in the colon, indicating enhanced microbial fermentation activity. This increased fermentation provides energy support to intestinal cells, thereby helping to maintain the stability of the intestinal barrier (75, 113). Moreover, SCFAs can improve the mechanical barrier function of the intestine by upregulating the expression of tight junction-related genes, consequently reducing intestinal permeability (114). In addition to SCFAs, bile acids, as bacterial metabolites, are also critical for maintaining the integrity of the intestinal barrier (115). Artificial milk has been shown to increase the protein expression of hepatic cholesterol 7α-hydroxylase (CYP7A1), reduce serum levels of conjugated bile acids, and enhance fecal bile acid excretion, thereby promoting bile acid metabolism (116) and ultimately supporting intestinal health in piglets (117).

Overall, in most cases, artificial milk can improve the health of the intestinal barrier by enriching SCFA-producing bacteria, enhancing SCFA and bile acid turnover, and exerting feedback effects on tight junction and mucin genes.

#### 4.2.3 Immune barrier

The intestine not only facilitates nutrient absorption but also houses the mucosal-associated lymphoid tissue (MALT) within the lamina propria, which constitutes the largest immune organ in the human body and plays a critical role in intestinal immune responses (118). This tissue promotes the initial activation and differentiation of immune cells (119) while also maintaining the integrity of immune cells and the epithelial barrier (120). Low body weight and insufficient immune competence adversely affect the growth performance and survival rate of piglets (121, 122). Therefore, efforts to improve growth performance in piglets should also prioritize the enhancement of their health and immune status (7).

Intestinal immune cells are capable of mounting both protective immune responses against pathogens and non-inflammatory reactions to dietary or bacterial antigens (123). Activation of these immune cells leads to the secretion of pro-inflammatory cytokines, which increase intestinal barrier permeability and thereby promote mucosal inflammation (124, 125). The roles of cytokines such as tumor necrosis factor-alpha (TNF-α), interleukin-1β (IL-1β), interleukin-6 (IL-6), and interleukin-8 (IL-8) in the intestinal barrier have attracted considerable attention. TNF-α, a pro-inflammatory cytokine involved in systemic inflammation, enhances intestinal epithelial barrier permeability through activation of the extracellular regulated protein kinases 1 and 2 signaling pathways (126). IL-1β stimulates T cells to secrete pro-inflammatory cytokines, induces chemokine production, influences B cell proliferation and macrophage activation, and systemically elevates body temperature and acute-phase protein levels (124, 125). IL-6 exerts pro-inflammatory effects by activating multiple target cells, including antigen-presenting cells and T cells (127). IL-8, a polymorphonuclear leukocyte chemotactic factor rapidly secreted by intestinal epithelial cells upon bacterial stimulation, initiates neutrophil migration to the mucosa (128) and is considered a marker of intestinal inflammation (129). Supplementation with artificial milk has been shown to reduce these pro-inflammatory cytokines and improve intestinal health. For instance, artificial milk supplementation significantly increased the mRNA level of Toll-like receptor 4 (TLR4) in the jejunum of 21-day-old piglets, while decreasing the relative mRNA levels of TNF-α and IL-8, which were negatively correlated with average daily gain. This reduction in inflammatory response may be attributed to enhanced jejunal epithelial cell proliferation and differentiation, improved intestinal metabolite and microbial composition, and upregulated expression of genes related to barrier and innate immune functions, ultimately improving growth performance (87). Furthermore, artificial milk supplementation also increased the relative mRNA expression of TLR4 and decreased IL-6 expression in the colonic mucosa of piglets (105). Similarly, piglets in the artificial milk group exhibited significantly lower mRNA expression levels of TNF-α, IL-1β, and IL-8, as well as reduced protein expression levels of TNF-α, IL-6, and IL-8 in the colonic mucosa (*p* < 0.05). Combined analysis of the intestinal microbiota composition suggested that the lower levels of inflammatory cytokines could be attributed to a reduction in the abundance of pro-inflammatory bacteria induced by artificial milk (79). Pro-inflammatory cytokines are associated with the development of intestinal inflammation (130), and the observed reduction in their expression following artificial milk supplementation indicates that artificial milk may enhance intestinal immune barrier health by attenuating intestinal inflammatory responses. Therefore, research on immune barriers indicates that supplementing with artificial milk reduces the expression levels of mucosal TNF-α, IL-1β, IL-6, and IL-8. These changes in cytokines subsequently decrease the body’s inflammatory response, thereby enhancing the health of the intestinal immune barrier.

In summary, at the level of intestinal morphology and digestive function, artificial milk promotes villus development and enhances brush border enzyme activity, thereby improving the capacity for nutrient digestion and absorption. Regarding intestinal barrier function, artificial milk optimizes the composition and metabolic products of the gut microbiota, strengthens the mechanical and mucus barriers, reduces the levels of pro-inflammatory cytokines, and mitigates inflammation. These changes collectively enhance intestinal barrier integrity and reinforce the protective role of the gut in the host organism.

## 5 Conclusion

Artificial milk serves as an important auxiliary tool in modern swine management, improving the survival rate of weak and supernumerary piglets, enhancing weaning weight and litter uniformity, alleviating the lactation burden on sows, and acting as a substitute when sow milk is insufficient. Additionally, artificial milk promotes intestinal development in piglets, thereby improving growth performance and facilitating the transition to solid feed after weaning. Future research should further investigate the combined effects of different formulations, supplementation strategies, and timing of artificial milk administration on the long-term growth and immune development of piglets, as well as on the reproductive performance of sows. Particular attention should be paid to the interactions among gut microbiota, digestive function, and behavioral management, in order to inform the management of sows with large litters and early-life rearing practices for piglets.
